# Genomic regulatory sequences in the pathogenesis of bipolar disorder

**DOI:** 10.3389/fpsyt.2023.1115924

**Published:** 2023-02-07

**Authors:** Anastasia Levchenko, Maria Plotnikova

**Affiliations:** ^1^Institute of Translational Biomedicine, Saint Petersburg State University, Saint Petersburg, Russia; ^2^Vavilov Institute of General Genetics, Russian Academy of Sciences, Moscow, Russia; ^3^Center for Genetics and Life Science, Sirius University of Science and Technology, Sochi, Russia

**Keywords:** bipolar disorder, genome-wide association study (GWAS), epigenetics, transcription, functional genetic variant, RNA

## Abstract

The lifetime prevalence of bipolar disorder is estimated to be about 2%. Epigenetics defines regulatory mechanisms that determine relatively stable patterns of gene expression by controlling all key steps, from DNA to messenger RNA to protein. This Mini Review highlights recent discoveries of modified epigenetic control resulting from genetic variants associated with bipolar disorder in genome-wide association studies. The revealed epigenetic abnormalities implicate gene transcription and post-transcriptional regulation. In the light of these discoveries, the Mini Review focuses on the genes *PACS1, MCHR1, DCLK3, HAPLN4, LMAN2L, TMEM258, GNL3, LRRC57, CACNA1C, CACNA1D*, and *NOVA2* and their potential biological role in the pathogenesis of bipolar disorder. Molecular mechanisms under control of these genes do not translate into a unified picture and substantially more research is needed to fill the gaps in knowledge and to solve current limitations in prognosis and treatment of bipolar disorder. In conclusion, the genetic and functional studies confirm the complex nature of bipolar disorder and indicate future research directions to explore possible targeted treatment options, eventually working toward a personalized approach.

## 1. Introduction

Bipolar disorder (BD) is viewed by clinicians as a spectrum of related, but differentiable conditions, spanning from BD type 1, to BD type 2, to cyclothymic disorder, to other specified or unspecified BD (1). In essence, BD type 1 is defined by the occurrence of at least one manic episode, BD type 2 is at least one hypomanic episode and at least one depressive episode, cyclothymic disorder is characterized by hypomanic and depressive symptoms lasting for at least 2 years that do not meet the full criteria for BD type 2, while other specified/unspecified BD do not meet the full criteria for the disorders mentioned above. The global lifetime prevalence of BD type 1 and type 2 is about 1% for each of these two diagnostic categories (1).

Epigenetics defines regulatory mechanisms that determine relatively stable patterns of gene expression by controlling all key steps, from DNA to messenger RNA (mRNA) to protein. These regulatory mechanisms, found to be implicated in the pathogenesis of many complex disorders (2, 3), including BD (4), function through DNA methylation, histone post-translational modifications (such as acetylation and methylation), and various levels of regulation of the 3D structure of chromatin (5). Abnormalities of some regulatory RNAs, like micro RNAs (miRNAs) (6) and long non-coding RNAs (7), also contribute to epigenetic mechanisms of complex disorders, including BD (8, 9). The epigenetic changes may be transmitted to next generations, but they are reversible (2, 4). Genomic variants can directly influence on epigenetics because of introduced differences in DNA sequences recognized by proteins and RNAs involved in epigenetic modifications; in this sense, there is an “interaction” between genomic variants and epigenetic mechanisms. However, epigenetic mechanisms are not always altered by these variants. Such a role of epigenetics in the pathogenesis of BD (i.e., no detectable involvement of changes in DNA) has been described elsewhere and the reader is referred to other reviews dealing with this topic (4, 10). The aim of the present Mini Review is to discuss epigenetic phenomena that result from interactions with genomic variants associated with BD (5). In particular, the article deals with common disease-associated variants discovered in genome-wide association studies (GWASs) and located in the same genomic loci than regulatory sequences implicated in: (a) regulation of gene transcription, (b) post-transcriptional regulation. The topics of pharmacogenetics and pharmacoepigenetics in BD are beyond the scope of the present article, so the reader is referred to other reviews covering these important topics (10–13).

## 2. Epigenetic consequences of genetic variants associated with BD

### 2.1. Overview of genetic architecture of BD

Genetic epidemiology studies estimated heritability of BD being in the range of 60–85% (14, 15). The clinical heterogeneity of BD (1) in fact parallels the genetic heterogeneity of this disorder (16–18). From the standpoint of both clinical manifestations and molecular biology, BD is a part of a spectrum where it forms a continuum with unipolar depression and schizophrenia (i.e., depression → BD type 2 → BD type 1 → schizophrenia) (19–23). Both common (22) and rare (24, 25) genetic variants contribute to the pathogenesis of BD: the first category explains ∼20% of the phenotypic variance, while the contribution of the second category is yet to be determined. The majority of the associated common variants are found in non-coding sequences of the genome and the reported rare variants change amino acid sequences of proteins.

GWASs harness common (≥1% minor allele frequency) genetic variants, traditionally referred to as single-nucleotide polymorphisms (SNPs), typed throughout the human genome to reveal associations between particular alleles of the variants and phenotypes in groups of unrelated individuals (26). The SNPs serve as markers: an associated SNP may have no functional role in itself, but it may be found in the same haplotype, i.e., be in linkage disequilibrium (LD) with another, this time functional variant. Coding sequences constitute only 1% of the human genome and *in silico* analyses indicate that the largest contribution to SNP heritability in complex traits comes from genetic variants in regulatory regions (27). GWASs of BD in particular, in comparison with the majority of other brain and non-brain disorders, show significant heritability enrichment in expression quantitative trait loci (eQTLs) and enhancers active in the brain, being second only to GWASs of schizophrenia (28, 29). An eQTL is a genetic variant whose alleles are associated with different quantities of a gene’s mRNA synthesized in different tissues. For example, according to the database GTEx (30), the variant rs4702 is an eQTL for the gene *FURIN* in the human frontal cortex where the reference allele G is associated with lower amounts of mRNA and the alternative allele A is associated with higher amounts of mRNA. Published data indicate that 65% of eQTLs are located in open chromatin at promoters and enhancers (5) and therefore regulate gene transcription.

The genetic studies mainly report results for BD type 1 and BD type 2 [e.g., (20, 22)]. The GWAS Catalog (31) lists 102 GWASs of BD (including types 1 and 2, cases with comorbidity, and cross-disorder cohorts, and excluding secondary phenotypes, such as therapeutic response), within 62 publications. These GWASs report 656 genome-wide significant associations (at a generally accepted *p*-value ≤ 5 × 10^–8^ that reflects the Bonferroni correction for testing associations with one million SNPs). A fraction of the associated SNPs were studied for their role in gene transcription and in post-transcriptional regulation. See Table 1 for the list of GWASs that reported these SNPs and Table 2 for the outline of functional studies, described in following sections, based on the GWASs.

**TABLE 1:** Genome-wide association studies (GWASs) used in the functional studies that investigated epigenetic alterations in bipolar disorder (BD).

GWAS of BD	Cases	Controls	Replication cases	Replication controls	Other details	Ancestry
Ferreira et al. (44)	4,387	6,209	–	–	–	European
Ruderfer et al. (45)	10,410 19,779 (BD + SCZ)	10,700 19,423 (BD + SCZ)	–	–	BD and SCZ cases were additionally combined	
Bipolar Disorder and Schizophrenia Working Group of the Psychiatric Genomics Consortium (34)	20,129 53,555 (BD + SCZ) 23,585 (SCZ) vs. 15,270 (BD)	21,524 54,065 (BD + SCZ)	–	–	BD and SCZ cases were additionally combined; also, SCZ cases were directly compared to BD cases	
Stahl et al. (20) (PGC2 GWAS)	20,352	31,358	9,412	137,760	–	
Mullins et al. (22) (PGC3 GWAS)	41,917	371,549	5,847	65,588	Polygenic risk scores based on GWAS were evaluated in samples of East Asian and admixed African American ancestries	

SCZ, schizophrenia.

**TABLE 2:** The outline of functional studies that revealed alterations in gene transcription and in post-transcriptional regulation linked to common genetic variants associated with bipolar disorder (BD).

Functional study	GWAS of BD	Implicated molecular mechanism	Type of regulatory sequences	Discovered coding genes
Gandal et al. (33)	Bipolar Disorder and Schizophrenia Working Group of the Psychiatric Genomics Consortium	Regulation of gene transcription	eQTLs	*BMPR1B, DCLK3, HAPLN4, HLF, LMAN2L, MCHR1, UBE2Q2L, SNAP91, TTC39A, TMEM258, VPS45*, and *NOVA2*
Hu et al. (38)	Stahl et al. (PGC2 GWAS)		Enhancers	–
Girdhar et al. (39)	Reference not indicated		Enhancers and *cis*-regulatory domains	–
Mullins et al. (22) (PGC3 GWAS)	Mullins et al. (PGC3 GWAS)		eQTLs	*STK4, ADD3, FURIN, HAPLN4, LMAN2L, PACSIN2, DCLK3, TMEM258, GNL3, PACS1, LRRC57, HTR6*, and *MCHR1*
Hall et al. (41)	Stahl et al. (PGC2 GWAS)		*Cis*-regulatory elements	*CDK10, CHMP1A, DDHD2, GLT8D1, GNL3, GOLGA6L4, GPN1, LRRC57, NMB, PACS1, SEMA3G*, and *XPNPEP3*
Chen et al. (43)	Stahl et al. (PGC2 GWAS)		Transcription factor binding sites	*NISCH, ZNF14, MTARC2, CILP2, SNX29P2, TMEM110, KRBOX1*, and *PACS1*
Eckart et al. (46)	Ferreira et al. and Ruderfer et al.		eQTLs	*CACNA1C*
Kouakou et al. (29)	Mullins et al. (PGC3 GWAS)		eQTLs, enhancers, and promoters	–
Park et al. (48)	Bipolar Disorder and Schizophrenia Working Group of the Psychiatric Genomics Consortium	Post-transcriptional regulation	RNA-binding proteins target sites	*ILF3*
Geaghan et al. (49)	Stahl et al. (PGC2 GWAS)		miRNA recognition elements (binding sites)	*NDUFA13, RBX1, NEK4, CACNA1D*, and *CHDH*

The GWASs that were used in the functional studies are detailed in Table 1.

### 2.2. Findings implicating regulation of gene transcription

The PsychENCODE Project has been making an invaluable contribution to our understanding of different levels of gene expression regulation in the human brain (28). The goal of the project is to produce “multidimensional genomic data using tissue- and cell type-specific samples from approximately 1,000 phenotypically well-characterized, high-quality healthy and disease-affected human post-mortem brains, as well as functionally characterize disease-associated regulatory elements and variants in model systems” (32). Several articles authored by the PsychENCODE Consortium reported findings in post-mortem brains of individuals with BD, some in connection with previous GWASs. The milestone study by Gandal et al. (33) investigated transcriptomic alterations in autism spectrum disorder, schizophrenia, and BD to discover disease- and cell type-specific changes in gene and isoform expression, by integrating the results in co-expression network modules. The study revealed that expression of the genes *BMPR1B, DCLK3, HAPLN4, HLF, LMAN2L, MCHR1, UBE2Q2L, SNAP91, TTC39A, TMEM258*, and *VPS45* is regulated by eQTLs (identified using human brain tissues) that are SNPs associated with BD in a GWAS reported by the Psychiatric Genomics Consortium (PGC) (34) [samples from the PGC2 GWAS of BD (20)]. These results in Gandal et al. were obtained by conducting a transcriptome-wide association study (TWAS) using the software FUSION (35), in addition to summary data-based Mendelian randomization (SMR) (36). In addition, eQTLs associated with BD were found to regulate expression of genes that interact in the network module “geneM7” active in excitatory neurons and enriched for synaptic processes (one of the top gene ontology terms is “synaptic vesicle cycle”) (33). Authors highlight the gene *NOVA2* in this network.

In a further investigation by the PsychENCODE Consortium the genome-wide chromosome conformation capture technique Hi-C (37) was applied in the postnatal human brain tissues from the dorsolateral prefrontal cortex to investigate chromosomal interactions between genomic regulatory elements in the context of different brain cell types, including glutamatergic and GABAergic neurons, and three brain disorders: Alzheimer’s disease, schizophrenia, and BD (38). Preliminary results show that genetic variants associated with BD in the PGC2 GWAS (20) are mostly present in enhancers active in the glutamatergic (excitatory) neurons of the upper cortical layers (cortical projection neurons and corticothalamic projection neurons), which differentiates this disorder from schizophrenia (38). However, both disorders are similar in terms of associations with enhancers active in GABAergic (inhibitory) parvalbumin-expressing interneurons (38).

A more recent study from the PsychENCODE Consortium harnessed chromatin immunoprecipitation followed by deep sequencing (ChIP-seq) using adult brain tissues from the prefrontal cortex of individuals with schizophrenia and BD, compared with controls, to investigate neuronal acetylation and methylation landscapes (39). The authors directed particular attention to *cis*-regulatory domains that are defined by histone epigenetic marks (peaks) and represent 3D organization of chromatin implicating various interacting *cis*-regulatory elements. The study used MAGMA (40) to discover that genomic regions containing hyper-acetylated histones that mark neuronal enhancers (H3K27ac) and hyper-acetylated *cis*-regulatory domains, all found in brain tissues of individuals with BD, contain SNPs associated with schizophrenia, but not with BD (39). The results could be explained by a combination of substantially smaller sample sizes in current GWASs of BD compared with GWASs of schizophrenia and substantial genetic correlation between schizophrenia and BD (21).

The most recent and the largest PGC3 GWAS of BD that included 41,917 cases and 371,549 controls also deployed FUSION to conduct a TWAS, followed by SMR, to reveal a transcriptomic impact of the BD-associated genomic loci (22). The results were based on eQTL data from the PsychENCODE Consortium (human post-mortem brains). The analysis revealed 13 coding genes, prioritized by FUSION and SMR as the most likely causal: *STK4, ADD3, FURIN, HAPLN4, LMAN2L, PACSIN2, DCLK3, TMEM258, GNL3, PACS1, LRRC57, HTR6*, and *MCHR1.*

Another study conducted TWASs of attention deficit hyperactivity disorder, autism spectrum disorder, major depressive disorder, schizophrenia, and BD to reveal *cis*-regulatory elements associated with these disorders (41). In particular, FUSION was used with summary statistics of the PGC2 GWAS of BD (20) to discover that genomic loci associated with BD contain fetal brain *cis*-regulatory elements for 22 genes, of which 12 are coding: *CDK10, CHMP1A, DDHD2, GLT8D1, GNL3, GOLGA6L4, GPN1, LRRC57, NMB, PACS1, SEMA3G*, and *XPNPEP3* (41). Authors highlight four of the coding genes–*NMB, LRRC57, DDHD2*, and *PACS1*–on the grounds of previous TWAS associations with BD (33, 42). *PACS1* stands out as the most convincing candidate gene (41).

A recent investigation was aimed at the identification of the functional SNPs in the reported BD risk loci and at revealing the regulatory mechanisms affected by these SNPs by deploying a battery of methods (43). Specifically, SNPs in LD with the 30 genome-wide significant SNPs from the PGC2 GWAS of BD (20) were integrated with ChIP-Seq data from human brain tissues or neuronal cell lines and with databases of eQTLs to determine transcription factor (TF) binding sequences that contain the associated SNPs. The identified functional SNPs were further validated using luciferase reporter assays in cell lines combined with TF gene silencing (with short hairpin RNAs) and deletions of functional SNPs (with CRISPR/Cas9). The authors revealed 16 functional SNPs that affect binding of TFs, mostly of CCCTC-binding factor (CTCF). There are seven coding genes significantly dysregulated in the brains of individuals with BD [data from PsychENCODE (33)] by the determined functional SNPs: *NISCH, ZNF14, MTARC2, CILP2, SNX29P2, TMEM110*, and *KRBOX1*. Importantly, this study confirmed the relevance of *PACS1* in the pathogenesis of BD: two SNPs rs10896081 and rs3862386 regulate the expression of *PACS1* and overexpression of this gene in mouse primary cortical neurons resulted in a reduced density of dendritic spines (43).

The variant rs1006737 associated with BD (44, 45) is an eQTL or is found in LD with an eQTL for *CACNA1C* (46). A study investigated the nature of this eQTL by testing 16 SNPs in high linkage disequilibrium with it (46). Authors used several approaches: measurements of mRNA expression in the superior temporal gyrus and of enhancer activity by luciferase reporter assays in neuroblastoma cell lines, as well as nuclear protein interaction assays and circular chromatin conformation capture and sequencing (4C-seq). The study suggested that rs4765905 is likely the functional variant in the superior temporal gyrus and the SK-N-SH neuroblastoma cell line.

Finally, open chromatin regions were tested for enrichment of SNPs associated with autism spectrum disorder, attention deficit hyperactivity disorder, major depressive disorder, schizophrenia, and BD (29). In this study, summary statistics from the PGC3 GWAS of BD (22) were integrated with results of the assay for transposase-accessible chromatin with high-throughput sequencing (ATAC-Seq) in the prenatal human frontal cortex (29). The authors discovered that SNPs associated with BD are in LD with eQTLs active in the human fetal frontal cortex and found in open chromatin regions with histone modifications indicative of enhancers (H3K4me1) and promoters (H3K4me3).

### 2.3. Findings implicating post-transcriptional regulation

RNA-binding proteins (RBPs) regulate the metabolism of RNAs, including the transcription, stability, splicing, localization, translation, and decay (47). Mounting evidence indicates that dysregulated RBP target sites are an important source of heritability of psychiatric disorders (48). A pioneering study investigated RBP target site dysregulation on the genome-wide scale in the context of psychiatric disorders (48). By leveraging the deep learning-based model Seqweaver (that predicts and quantifies RBP target site dysregulation) and the LD score regression statistical method, the study determined RBP target sites in the human genome enriched in SNPs associated with attention deficit hyperactivity disorder, autism spectrum disorder, major depressive disorder, schizophrenia, and BD (48). Specifically, by using summary statistics from one of the PGC’s GWASs of BD (34), the authors showed that the RBP target sites in BD are significantly dysregulated. Furthermore, the effect size of genetic variants associated with BD in the target site for the RBP interleukin enhancer-binding factor 3 (ILF3) was found to be significantly greater than for schizophrenia (48). The regulation of expression of this RBP was also significantly associated with BD, but not with schizophrenia in the study by Gandal et al. (33).

Another epigenetic component miRNAs regulate the stability of mRNAs through the mechanism of RNA interference and thus are implicated in post-transcriptional regulation (6, 9). Their binding sites called miRNA recognition elements (MREs) are often found in 3′ untranslated regions (3′-UTRs) of genes. An investigation of the relevance of MREs in genomic loci associated with schizophrenia, major depressive disorder, autism spectrum disorder, post-traumatic stress disorder, attention deficit hyperactivity disorder, anorexia nervosa, obsessive compulsive disorder, Tourette’s syndrome, and BD was conducted (49). In this study, authors used summary statistics of the PGC2 GWAS of BD (20) and a multi-step bioinformatic analysis to reveal that the genetic variants significantly associated with BD affect a number of MREs, including those that coincide with GTEx eQTLs active in the brain. Authors also determined four associated miRNA families: miR-151-5p, miR-409-5p, miR-218-5p, and miR-212-5p. Moreover, the MREs with variants associated with BD are found in five coding genes, as was determined by MAGMA (40): *NDUFA13, RBX1, NEK4, CACNA1D*, and *CHDH* (49).

## 3. Discussion

This Mini Review highlighted recent discoveries of modified epigenetic control resulting from genetic variants associated with BD. Eight coding genes revealed by at least two studies and other three coding genes with functional connections in multiple studies deserve further discussion of their possible biological roles in the pathogenesis of BD.

*PACS1* reported in three studies (22, 41, 43) codes for Phosphofurin Acidic Cluster Sorting Protein 1. The functional role of this gene was confirmed not only with transcriptome-based data, but also with a cell model where its overexpression resulted in a reduced density of dendrites. The encoded protein is implicated in localization of other proteins in the post-synaptic trans-Golgi network (42). It interacts in particular with FURIN, Paired Basic Amino Acid Cleaving Enzyme, also reported in the PGC3 GWAS of BD (22). PASC1 has the amino acid substitution R203W in rare cases of syndromic intellectual disability (50). Overall, these data indicate that PACS1 could be particularly important post-synaptically by contributing to the maintenance of dendrites.

*MCHR1, DCLK3, HAPLN4, LMAN2L*, and *TMEM258* (22, 33), as well as *GNL3* and *LRRC57* (22, 41) were each reported by two studies that used similar approaches based on transcriptome data. *MCHR1* codes for Melanin Concentrating Hormone Receptor 1 that is a G protein-coupled receptor mainly expressed in the hypothalamus (51). This receptor controls a wide range of body functions, including feeding, energy expenditure, locomotion, sleep-wake cycle, and sexual, exploratory, and aggressive behaviors (51). All these domains are compromised in BD (52). *DCLK3* that codes for Doublecortin Like Kinase 3 is expressed in the striatum and hippocampus; it is involved in regulation of transcription and has a neuroprotective role (53). Reduced volumes of the hippocampus are one of the neurobiological findings in BD (54). In addition, the encoded protein is deemed druggable (22). Finally, *GNL3* codes for G Protein Nucleolar 3, a protein that promotes cell proliferation in neural stem and progenitor cells and prevents subsequent neuronal differentiation (55, 56). *GNL3*’s expression levels are increased in BD (33). SNPs in this gene associated with BD are also associated with volumes of numerous structures of the human brain, including basal ganglia (56). Therefore, the role of *GNL3* in the pathogenesis of BD could be linked to the neurodevelopmental aspects of BD (57).

The roles of *HAPLN4, LMAN2L*, *TMEM258*, and *LRRC57* in the pathogenesis of BD are less obvious. *HAPLN4* codes for Hyaluronan and Proteoglycan Link Protein 4 that secures proper neuronal communication in the cerebellum (58). *LMAN2L* encodes Lectin, Mannose Binding 2 Like that guides certain glycoproteins from the endoplasmic reticulum to extracellular secretion (59). Pathogenic genetic variants in this gene are associated with syndromic intellectual disability (59). The product of *TMEM258*, Transmembrane Protein 258, is involved in N-linked protein glycosylation and regulation of stress responses in the endoplasmic reticulum (60). The function of the gene *LRRC57*, coding for Leucine Rich Repeat Containing 57, is still obscure.

*CACNA1C* (46) and *CACNA1D* (49) code for Calcium Voltage-Gated Channel Subunits Alpha1 C and D, respectively. In the context of the present Mini Review, the implication of the two genes in the pathogenesis of BD was determined based on publications that used quite different approaches revealing changes in transcriptional and post-transcriptional regulation of these genes brought about by genetic variants associated with BD. However, the two genes are tightly related functionally. These subunits are used to make the “pore” of calcium voltage-gated channels present on a number of cell types. The channels are also present on neurons where they regulate neurotransmitter release: when the depolarization of the action potential arrives at the axonal terminal, the pore lets the calcium ions enter the pre-synaptic neuron which causes a release of synaptic vesicles containing a neurotransmitter into the synaptic cleft (61). Importantly, these channels are also implicated in neurodevelopment (62) and neuroplasticity through gene expression regulation (63, 64). Entering Ca^2+^ binds to calmodulin attached to the cytoplasmic portion of the channel (64). This binding activates the Ras/mitogen-activated protein kinase (MAPK) pathway that results in recruitment of transcription factors, such as cyclic adenosine monophosphate response element binding protein (CREB), that induce the expression of genes important in neuroplasticity (64). Additional evidence indicates that the activity of this channel is necessary for adult hippocampal neurogenesis (63). These findings offer an explanation for the observed brain structural abnormalities in BD (65), which could be caused by deficient adult neurogenesis (54) or by abnormal neurodevelopment (57). In support of these findings, missense variants in *CACNA1C* and *CACNA1D* are associated with syndromic intellectual disability (66) and common variants are associated with psychiatric disorders (63, 67).

Finally, *NOVA2* reported by Gandal et al. (33) codes for Neuro-Oncological Ventral Antigen 2, a neuronal splicing regulator (68). In the light of the emerging evidence of dysregulated RBP target sites in the pathogenesis of psychiatric disorders (48), regulation of mRNA splicing could be another biological mechanism that contributes significantly to the pathogenesis of BD.

In conclusion, functional investigations of the regulatory genetic variants associated with BD confirm the substantially complex nature of this psychiatric disorder. Both pre- and post-synaptic components, as well as neurodevelopmental and neuroprotective aspects contribute to the pathogenesis. These distinct elements do not translate into a clear unified picture and substantially more research is needed to fill the gaps in knowledge and to solve current limitations in prognosis and treatment of BD. Multiple avenues should be pursued, including further efforts in the study of epigenetic consequences of BD-associated genetic variants. For example, to increase the rate of discovery of functional common genetic variants, larger cohorts with more detailed phenotypes should be used to perform GWASs. Detailed phenotyping will allow identifying more homogeneous groups of patients with the hope that similarities in phenotypes are a reflection of similar pathogenic mechanisms involved. Likewise, more attention should be given to rare, high impact genetic variants associated with BD, such as single nucleotide substitutions, indels, or copy number variants (24, 25), with the hope that studying their biological consequences is relatively straightforward (compared to common variants), which will speed up downstream discoveries. The complex biology of BD, despite creating obvious challenges for researches, could on the other hand allow exploring different possible targeted treatment options for the patients. Perhaps, our goal should be identifying the main relevant molecular pathways compromised in different groups of patients, rather than searching for one common pathogenic mechanism of BD. Such an approach will constitute first steps toward personalized psychiatry (69, 70).

## Author contributions

AL contributed to the conception of the mini-review and prepared the first draft. AL and MP wrote sections of the manuscript. Both authors contributed to the manuscript revision, read, and approved the submitted version.

## References

[1] McIntyre R Berk M Brietzke E Goldstein B Lopez-Jaramillo C Kessing L Bipolar Disorders. *Lancet.* (2020) 396:1841–56. 10.1016/S0140-6736(20)31544-0 33278937

[2] Schiele M Gottschalk M Domschke K. The applied implications of epigenetics in anxiety, affective and stress-related disorders - a review and synthesis on psychosocial stress, psychotherapy and prevention. *Clin Psychol Rev.* (2020) 77:101830. 10.1016/j.cpr.2020.101830 32163803

[3] Richetto J Meyer U. Epigenetic modifications in schizophrenia and related disorders: molecular scars of environmental exposures and source of phenotypic variability. *Biol Psychiatry.* (2021) 89:215–26. 10.1016/j.biopsych.2020.03.008 32381277

[4] Ludwig B Dwivedi Y. Dissecting bipolar disorder complexity through epigenomic approach. *Mol Psychiatry.* (2016) 21:1490–8. 10.1038/mp.2016.123 27480490 PMC5071130

[5] Powell S O’Shea C Brennand K Akbarian S. Parsing the functional impact of noncoding genetic variants in the brain epigenome. *Biol Psychiatry.* (2021) 89:65–75. 10.1016/j.biopsych.2020.06.033 33131715 PMC7718420

[6] Leitao A Enguita F. A structural view of mirna biogenesis and function. *Non-coding RNA.* (2022) 8:10. 10.3390/ncrna8010010 35202084 PMC8874510

[7] Engreitz J Ollikainen N Guttman M. Long Non-Coding Rnas: Spatial amplifiers that control nuclear structure and gene expression. *Nat Rev Mol Cell Biol.* (2016) 17:756–70. 10.1038/nrm.2016.126 27780979

[8] Bella F Campo S. Long Non-Coding Rnas and Their Involvement in Bipolar Disorders. *Gene.* (2021) 796-797:145803. 10.1016/j.gene.2021.145803 34175394

[9] Fries G Carvalho A Quevedo J. The mirnome of bipolar disorder. *J Affect Disord.* (2018) 233:110–6. 10.1016/j.jad.2017.09.025 28969861

[10] Legrand A Iftimovici A Khayachi A Chaumette B. Epigenetics in Bipolar Disorder: A critical review of the literature. *Psychiatr Genet.* (2021) 31:1–12. 10.1097/YPG.0000000000000267 33290382

[11] Vecera C Fries G Shahani L Soares J Machado-Vieira R. Pharmacogenomics of lithium response in bipolar disorder. *Pharmaceuticals (Basel).* (2021) 14:287. 10.3390/ph14040287 33804842 PMC8063790

[12] Papiol S Schulze T Heilbronner U. Lithium response in bipolar disorder: Genetics, genomics, and beyond. *Neurosci Lett.* (2022) 785:136786. 10.1016/j.neulet.2022.136786 35817312

[13] Pisanu C Meloni A Severino G Squassina A. Genetic and epigenetic markers of lithium response. *Int J Mol Sci.* (2022) 23:1555. 10.3390/ijms23031555 35163479 PMC8836013

[14] Smoller J Finn C. Family, twin, and adoption studies of bipolar disorder. *Am J Med Genet C Semin Med Genet.* (2003) 123C:48–58. 10.1002/ajmg.c.20013 14601036

[15] Johansson V Kuja-Halkola R Cannon T Hultman C Hedman A. A population-based heritability estimate of bipolar disorder - in a swedish twin sample. *Psychiatry Res.* (2019) 278:180–7. 10.1016/j.psychres.2019.06.010 31207455

[16] Coombes B Markota M Mann J Colby C Stahl E Talati A Dissecting clinical heterogeneity of bipolar disorder using multiple polygenic risk scores. *Transl Psychiatry.* (2020) 10:314. 10.1038/s41398-020-00996-y 32948743 PMC7501305

[17] Gordovez F McMahon F. The genetics of bipolar disorder. *Mol Psychiatry.* (2020) 25:544–59. 10.1038/s41380-019-0634-7 31907381

[18] Richards A Cardno A Harold G Craddock N Di Florio A Jones L Genetic liabilities differentiating bipolar disorder, schizophrenia, and major depressive disorder, and phenotypic heterogeneity in bipolar disorder. *JAMA Psychiatry.* (2022) 79:1032–9. 10.1001/jamapsychiatry.2022.2594 36044200 PMC9434480

[19] Gandal M Haney J Parikshak N Leppa V Ramaswami G Hartl C Shared molecular neuropathology across major psychiatric disorders parallels polygenic overlap. *Science.* (2018) 359:693–7. 10.1126/science.aad6469 29439242 PMC5898828

[20] Stahl E Breen G Forstner A McQuillin A Ripke S Trubetskoy V Genome-Wide association study identifies 30 loci associated with bipolar disorder. *Nat Genet.* (2019) 51:793–803. 10.1038/s41588-019-0397-8 31043756 PMC6956732

[21] Cross-Disorder Group of the Psychiatric Genomics Consortium. Genomic relationships, novel loci, and pleiotropic mechanisms across eight psychiatric disorders. *Cell.* (2019) 179:1469–82. 10.1016/j.cell.2019.11.020 31835028 PMC7077032

[22] Mullins N Forstner A O’Connell K Coombes B Coleman J Qiao Z Genome-Wide association study of more than 40,000 bipolar disorder cases provides new insights into the underlying biology. *Nat Genet.* (2021) 53:817–29. 10.1038/s41588-021-00857-4 34002096 PMC8192451

[23] Coleman J Gaspar H Bryois J Bipolar Disorder Working Group of the Psychiatric Genomics Consortium, Major Depressive Disorder Working Group of the Psychiatric Genomics Consortium, Breen G. The genetics of the mood disorder spectrum: genome-wide association analyses of more than 185,000 cases and 439,000 controls. *Biol Psychiatry.* (2020) 88:169–84. 10.1016/j.biopsych.2019.10.015 31926635 PMC8136147

[24] Palmer D Howrigan D Chapman S Adolfsson R Bass N Blackwood D Exome sequencing in bipolar disorder identifies akap11 as a risk gene shared with schizophrenia. *Nat Genet.* (2022) 54:541–7. 10.1038/s41588-022-01034-x 35410376 PMC9117467

[25] Kushima I Nakatochi M Aleksic B Okada T Kimura H Kato H Cross-Disorder analysis of genic and regulatory copy number variations in bipolar disorder, schizophrenia, and autism spectrum disorder. *Biol Psychiatry.* (2022) 92:362–74. 10.1016/j.biopsych.2022.04.003 35667888

[26] Uffelmann E Huang Q Munung N de Vries J Okada Y Martin A Genome-Wide Association Studies. *Nat Rev Methods Primers.* (2021) 1:59. 10.1038/s43586-021-00056-9

[27] Gusev A Lee S Trynka G Finucane H Vilhjalmsson B Xu H Partitioning heritability of regulatory and cell-type-specific variants across 11 common diseases. *Am J Hum Genet.* (2014) 95:535–52. 10.1016/j.ajhg.2014.10.004 25439723 PMC4225595

[28] Wang D Liu S Warrell J Won H Shi X Navarro F Comprehensive functional genomic resource and integrative model for the human brain. *Science.* (2018) 362:eaat8464. 10.1126/science.aat8464 30545857 PMC6413328

[29] Kouakou M Cameron D Hannon E Dempster E Mill J Hill M Sites of active gene regulation in the prenatal frontal cortex and their role in neuropsychiatric disorders. *Am J Med Genet B Neuropsychiatr Genet.* (2021) 186:376–88. 10.1002/ajmg.b.32877 34632689 PMC7619043

[30] GTEx Consortium. The gtex consortium atlas of genetic regulatory effects across human tissues. *Science.* (2020) 369:1318–30. 10.1126/science.aaz1776 32913098 PMC7737656

[31] Buniello A MacArthur J Cerezo M Harris L Hayhurst J Malangone C The Nhgri-Ebi Gwas Catalog of Published Genome-Wide Association Studies, Targeted Arrays and Summary Statistics 2019. *Nucleic Acids Res.* (2019) 47:D1005–12. 10.1093/nar/gky1120 30445434 PMC6323933

[32] PsychEncode Consortium Akbarian S Liu C Knowles J Vaccarino F Farnham P The Psychencode Project. *Nat Neurosci.* (2015) 18:1707–12. 10.1038/nn.4156 26605881 PMC4675669

[33] Gandal M Zhang P Hadjimichael E Walker R Chen C Liu S Transcriptome-Wide isoform-level dysregulation in asd, schizophrenia, and bipolar disorder. *Science.* (2018) 362:eaat8127. 10.1126/science.aat8127 30545856 PMC6443102

[34] Bipolar Disorder and Schizophrenia Working Group of the Psychiatric Genomics Consortium. Genomic dissection of bipolar disorder and schizophrenia, including 28 subphenotypes. *Cell.* (2018) 173:1705–15. 10.1016/j.cell.2018.05.046 29906448 PMC6432650

[35] Gusev A Ko A Shi H Bhatia G Chung W Penninx B Integrative approaches for large-scale transcriptome-wide association studies. *Nat Genet.* (2016) 48:245–52. 10.1038/ng.3506 26854917 PMC4767558

[36] Zhu Z Zhang F Hu H Bakshi A Robinson M Powell J Integration of summary data from gwas and eqtl studies predicts complex trait gene targets. *Nat Genet.* (2016) 48:481–7. 10.1038/ng.3538 27019110

[37] van Berkum N Lieberman-Aiden E Williams L Imakaev M Gnirke A Mirny L Hi-C: A Method to Study the Three-Dimensional Architecture of Genomes. *J Vis Exp.* (2010) 6:1869. 10.3791/1869 20461051 PMC3149993

[38] Hu B Won H Mah W Park R Kassim B Spiess K Neuronal and Glial 3d chromatin architecture informs the cellular etiology of brain disorders. *Nat Commun.* (2021) 12:3968. 10.1038/s41467-021-24243-0 34172755 PMC8233376

[39] Girdhar K Hoffman G Bendl J Rahman S Dong P Liao W Chromatin domain alterations linked to 3d genome organization in a large cohort of schizophrenia and bipolar disorder brains. *Nat Neurosci.* (2022) 25:474–83. 10.1038/s41593-022-01032-6 35332326 PMC8989650

[40] de Leeuw C Mooij J Heskes T Posthuma D. Magma: Generalized gene-set analysis of gwas data. *PLoS Comput Biol.* (2015) 11:e1004219. 10.1371/journal.pcbi.1004219 25885710 PMC4401657

[41] Hall L Pain O O’Brien H Anney R Walters J Owen M Cis-Effects on gene expression in the human prenatal brain associated with genetic risk for neuropsychiatric disorders. *Mol Psychiatry.* (2021) 26:2082–8. 10.1038/s41380-020-0743-3 32366953 PMC7611670

[42] Huckins L Dobbyn A McFadden W Wang W Ruderfer D Hoffman G Transcriptomic imputation of bipolar disorder and bipolar subtypes reveals 29 novel associated genes. *bioRxiv* [preprint]. (2017): 10.1101/222786

[43] Chen R Yang Z Liu J Cai X Huo Y Zhang Z Functional genomic analysis delineates regulatory mechanisms of gwas-identified bipolar disorder risk variants. *Genome Med.* (2022) 14:53. 10.1186/s13073-022-01057-3 35590387 PMC9121601

[44] Ferreira M O’Donovan M Meng Y Jones I Ruderfer D Jones L Collaborative genome-wide association analysis supports a role for ank3 and cacna1c in bipolar disorder. *Nat Genet.* (2008) 40:1056–8. 10.1038/ng.209 18711365 PMC2703780

[45] Ruderfer D Fanous A Ripke S McQuillin A Amdur R Schizophrenia Working Group of the Psychiatric Genomics Consortium Polygenic Dissection of Diagnosis and Clinical Dimensions of Bipolar Disorder and Schizophrenia. *Mol Psychiatry.* (2014) 19:1017–24. 10.1038/mp.2013.138 24280982 PMC4033708

[46] Eckart N Song Q Yang R Wang R Zhu H McCallion A Functional characterization of schizophrenia-associated variation in cacna1c. *PLoS One.* (2016) 11:e0157086. 10.1371/journal.pone.0157086 27276213 PMC4898738

[47] Gebauer F Schwarzl T Valcarcel J Hentze M. Rna-Binding proteins in human genetic disease. *Nat Rev Genet.* (2021) 22:185–98. 10.1038/s41576-020-00302-y 33235359

[48] Park C Zhou J Wong A Chen K Theesfeld C Darnell R Genome-Wide landscape of rna-binding protein target site dysregulation reveals a major impact on psychiatric disorder risk. *Nat Genet.* (2021) 53:166–73. 10.1038/s41588-020-00761-3 33462483 PMC7886016

[49] Geaghan M Reay W Cairns M. Microrna binding site variation is enriched in psychiatric disorders. *Hum Mutat.* (2022) 43:2153–69. 10.1002/humu.24481 36217923 PMC10947041

[50] Van Nuland A Reddy T Quassem F Vassalli J Berg A. Pacs1-Neurodevelopmental Disorder: Clinical Features and Trial Readiness. *Orphanet J Rare Dis.* (2021) 16:386. 10.1186/s13023-021-02001-1 34517877 PMC8438988

[51] Presse F Conductier G Rovere C Nahon J. The melanin-concentrating hormone receptors: Neuronal and non-neuronal functions. *Int J Obesity Suppl.* (2014) 4(Suppl 1):S31–6. 10.1038/ijosup.2014.9 27152164 PMC4850588

[52] Vieta E Berk M Schulze T Carvalho A Suppes T Calabrese J Bipolar Disorders. *Nat Rev Dis Primers.* (2018) 4:18008. 10.1038/nrdp.2018.8 29516993

[53] Galvan L Francelle L Gaillard M de Longprez L Carrillo-de Sauvage M Liot G The striatal kinase dclk3 produces neuroprotection against mutant huntingtin. *Brain.* (2018) 141:1434–54. 10.1093/brain/awy057 29534157 PMC5917821

[54] Chepenik L Wang F Spencer L Spann M Kalmar J Womer F Structure-function associations in hippocampus in bipolar disorder. *Biol Psychol.* (2012) 90:18–22. 10.1016/j.biopsycho.2012.01.008 22342942 PMC3319637

[55] Tsai R McKay R. A nucleolar mechanism controlling cell proliferation in stem cells and cancer cells. *Genes Dev.* (2002) 16:2991–3003. 10.1101/gad.55671 12464630 PMC187487

[56] Meng Q Wang L Dai R Wang J Ren Z Liu S Integrative analyses prioritize gnl3 as a risk gene for bipolar disorder. *Mol Psychiatry.* (2020) 25:2672–84. 10.1038/s41380-020-00866-5 32826963

[57] Kloiber S Rosenblat J Husain M Ortiz A Berk M Quevedo J Neurodevelopmental pathways in bipolar disorder. *Neurosci Biobehav Rev.* (2020) 112:213–26. 10.1016/j.neubiorev.2020.02.005 32035092

[58] Edamatsu M Miyano R Fujikawa A Fujii F Hori T Sakaba T Hapln4/Bral2 Is a selective regulator for formation and transmission of gabaergic synapses between purkinje and deep cerebellar nuclei neurons. *J Neurochem.* (2018) 147:748–63. 10.1111/jnc.14571 30125937

[59] Alkhater R Wang P Ruggieri A Israelian L Walker S Scherer S Dominant Lman2l mutation causes intellectual disability with remitting epilepsy. *Ann Clin Transl Neurol.* (2019) 6:807–11. 10.1002/acn3.727 31020005 PMC6469342

[60] Graham D Lefkovith A Deelen P de Klein N Varma M Boroughs A Tmem258 Is a component of the oligosaccharyltransferase complex controlling Er stress and intestinal inflammation. *Cell Rep.* (2016) 17:2955–65. 10.1016/j.celrep.2016.11.042 27974209 PMC5661940

[61] Atlas D. The voltage-gated calcium channel functions as the molecular switch of synaptic transmission. *Annu Rev Biochem.* (2013) 82:607–35. 10.1146/annurev-biochem-080411-121438 23331239

[62] Smedler E Louhivuori L Romanov R Masini D Dehnisch Ellstrom I Wang C Disrupted Cacna1c Gene Expression Perturbs Spontaneous Ca(2+) Activity Causing Abnormal Brain Development and Increased Anxiety. *Proc Natl Acad Sci USA.* (2022) 119:e2108768119. 10.1073/pnas.2108768119 35135875 PMC8851547

[63] Moon A Haan N Wilkinson L Thomas K Hall J. Cacna1c: Association with psychiatric disorders, behavior, and neurogenesis. *Schizophr Bull.* (2018) 44:958–65. 10.1093/schbul/sby096 29982775 PMC6101623

[64] Dolmetsch R Pajvani U Fife K Spotts J Greenberg M. Signaling to the Nucleus by an L-Type Calcium Channel-Calmodulin Complex through the Map Kinase Pathway. *Science.* (2001) 294:333–9. 10.1126/science.1063395 11598293

[65] Houenou J d’Albis M Vederine F Henry C Leboyer M Wessa M. Neuroimaging biomarkers in bipolar disorder. *Front Biosci (Elite Ed).* (2012) 4:593–606. 10.2741/e402 22201897

[66] Kessi M Chen B Peng J Yan F Yang L Yin F. Calcium channelopathies and intellectual disability: A systematic review. *Orphanet J Rare Dis.* (2021) 16:219. 10.1186/s13023-021-01850-0 33985586 PMC8120735

[67] Amare A Schubert K Klingler-Hoffmann M Cohen-Woods S Baune B. The genetic overlap between mood disorders and cardiometabolic diseases: A systematic review of genome wide and candidate gene studies. *Transl Psychiatry.* (2017) 7:e1007. 10.1038/tp.2016.261 28117839 PMC5545727

[68] Mattioli F Hayot G Drouot N Isidor B Courraud J Hinckelmann M De Novo Frameshift Variants in the Neuronal Splicing Factor Nova2 Result in a Common C-Terminal Extension and Cause a Severe Form of Neurodevelopmental Disorder. *Am J Hum Genet.* (2020) 106:438–52. 10.1016/j.ajhg.2020.02.013 32197073 PMC7118572

[69] Levchenko A Nurgaliev T Kanapin A Samsonova A Gainetdinov R. Current challenges and possible future developments in personalized psychiatry with an emphasis on psychotic disorders. *Heliyon.* (2020) 6:e03990. 10.1016/j.heliyon.2020.e03990 32462093 PMC7240336

[70] Alhajji L Nemeroff C. Personalized medicine and mood disorders. *Psychiatr Clin North Am.* (2015) 38:395–403. 10.1016/j.psc.2015.05.003 26300030

